# Korean primary health care program for people with disabilities: do they really want home-based primary care?

**DOI:** 10.1186/s12913-023-10102-9

**Published:** 2023-10-11

**Authors:** Hye-Jin Kim, Jae-Young Lim, Soong-Nang Jang

**Affiliations:** 1 Red Cross College of Nursing, Chung-Ang University, 84 Heukseok-Ro, Dongjak-Gu, Seoul, 06974 South Korea; 2 Department of Gyeonggi Regional Health & Medical Center for Persons with Disabilities, Seoul National University Bundang Hospital, Seongnam, Gyeonggi Korea; 3 Department of Rehabilitation Medicine, Seoul National University College of Medicine, Seoul National University Bundang Hospital, Seongnam, Gyeonggi Korea

**Keywords:** Home-Based Primary Care, Disabilities, Healthcare access, Health services for people with disabilities, Service demand

## Abstract

**Background:**

Despite many studies on home-based primary care (HBPC)-related benefits and challenges, little is known about the perspectives of potential target groups of the care and their intention or preference for using it. This study aimed to explore the demand for HBPC from the perspective of people with disabilities (PWDs) and caregivers and identify relevant determinants for that demand.

**Methods:**

Data from the population-based survey conducted in the Gyeonggi Regional Health & Medical Center for People with Disabilities in South Korea were analyzed. Logistic regression analysis was performed to identify relevant determinants for the demand on HBPC.

**Results:**

Overall, 22% of respondents required HBPC, and 34.7% of persons aged ≥ 65 years demanded it. Older adults with disability, homebound status, and a need for assistance with daily living activities were associated with a demand for HBPC. Though having severe disability, only 19.49% of self-reported respondents demanded for HBPC, while 39.57% of proxy-reported respondents demanded for HBPC. Among self-reported group, only marital status was a predictor associated with a demand for HBPC. In contrast, among proxy-reported groups, PWDs with external physical disabilities, or with unmet medical needs due to availability barriers reported a higher demand for HBPC.

**Conclusions:**

The demand for HBPC does not derive from the medical demands of the users themselves, but rather the care deficit by difficulty in getting out of the house or in outpatient care. Beyond an alternative to office-based care, HBPC needs to be considered to solve the care deficit and as well as to deal with PWDs’ medical problems.

## Background

Home-based primary care (HBPC) is recognized as a suitable model to vulnerable populations (e.g., persons with disabilities [PWDs]) who have high medical needs due to multiple chronic conditions but have barriers to accessing to office-based care [1–4]. It reportedly reduces hospitalizations, rehospitalizations, emergency department (ED) visits, admissions for long-term care, mortality, functional decline, medical cost saving, and caregiver burden, thereby improving individual and caregiver quality of life and satisfactions [3, 5–7]. However, most HBPC programs have emerged for homebound older adults or Medicare beneficiaries [5]. Only a few studies focusing on PWDs reported positive impacts, such as decreasing hospitalization rates in patients with intellectual and/or developmental disability (IDD) [8] or ED and hospital use in PWDs aged ≥ 18 years, posing substantial barriers to office-based primary care [4].

Based on the positive evidence, HBPC have received much attention. In the US, HBPC programs have been delivered primarily by Department of Veterans Affairs (VA) and the Medicare Independence at Home Project [9], and family physicians provides HBPC to patients such as homebound patients in Canada, but providing home visits is not a requirement [10]. Moreover, preventive home visitation programs for medically and socially vulnerable elderly people are part of national policy in Denmark, and Australia [11, 12]. In South Korea, according to the revised version of the National Health Insurance Act in December 2018, for the first time in Korean history, physicians have been allowed to visit patients’ homes if the patient has a severe disabling condition or special needs such as hospice care [13, 14]. Since then, several HBPC pilot projects have been planned by the central government, such as a HBPC pilot program for homebound older adults with long-term care needs [14], HBPC for PWDs, and HBPC for patients with difficulties for office-based care (i.e., patients with ulcer or mental disorders).

Among them, HBPC for PWDs is a newly attempted service under the primary healthcare (PHC) pilot program by the central government. Since the enactment of the Right to Health and Access to Medical Services for Persons with Disabilities Act [15], the government launched a PHC pilot program for PWDs in 2018. This program involves team-based healthcare and HBPC for people with registered severe disabilities [16]. The government has attempted to improve this PHC initiative for PWD since its launch. The third PHC initiative for PWD (1st project, May 2018–May 2020; 2nd project, June 2020–September 29, 2021) includes a comprehensive health assessment and care planning (compulsory), mid-term management (optional), education/consultation (optional), patient monitoring (optional), doctor or nurse visits (optional), and a voucher for a health check-up (optional). The cost is fee-for-services based; PWD who use the service pay 10% of the cost (0% co-payment for some services, such as health assessments), while the government pays the remaining 90% of the cost.

Although people with registered severe disability can participate in the pilot project and receive the above benefits from participating PHC clinicians, as of December 2020 only 0.09% of the potential service users (984,965 people with registered severe disabilities in South Korea) had participated in the pilot program; This program has been underused because of low awareness, poor demand incentives, and supply structure [17]. Some physicians who participated in the program reported difficulties in finding the patients preferring the HBPC program [18]. In contrast, most PWDs and their caregivers were not aware of this system. In addition, the physical, economic, and psychological accessibility was quite low, and care services that did not reflect PWD-specific needs were reported as problems [17, 19]. On the service consumer’s perspective, the main caregiver’s opinions need to be identified as much as those of PWDs, since the high caregiver burden as a social issue could be alleviated through HBPC [6]. Moreover, a dynamic triangular interaction among the patient, caregiver, and physician enables patient-centered primary care in some populations, such as people with IDD [20].

Efforts to track patients’ demands across care settings can improve healthcare delivery and reduce the likelihood of unmet healthcare service needs [17]. There is a need to explore the demands for HBPC, specifically from the perspective of PWDs and caregivers. However, despite many studies on HBPC-related benefits and challenges, little is known about the perspectives of potential target groups of HBPC and their intention or preference for using HBPC. There was a lack of detailed understanding on how much and under what circumstances PWDs requested for HBPC.

**Fig. 1:**
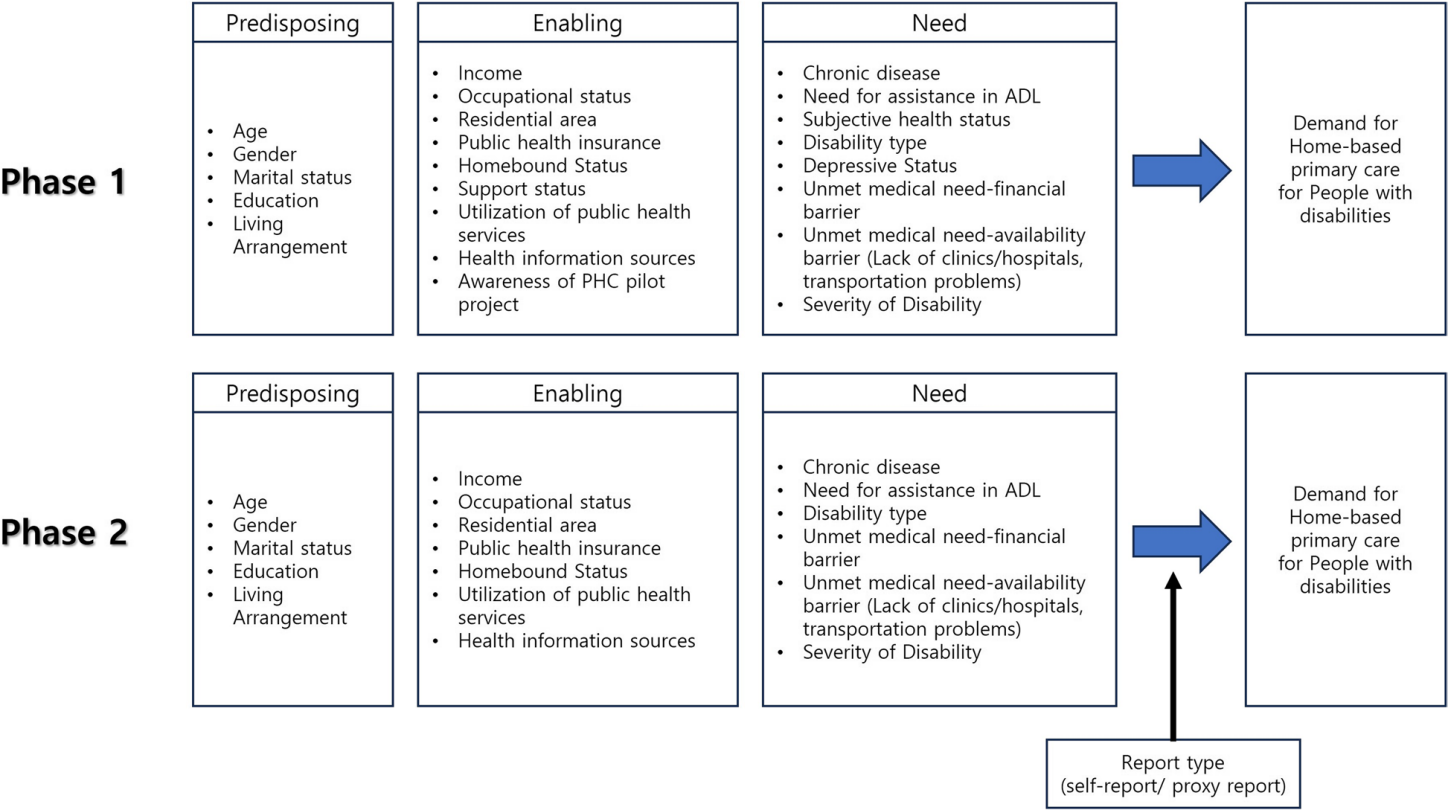
Study framework

Thus, our study was designed as two-phase to describe demands for HBPC for PWDs among a large population-based sample and identify relevant determinants for that demand using Andersen’s behavioral model of health service use (Fig. 1). This model is widely used to analyze factors affecting unmet healthcare needs, as it is designed to consider individual and environmental factors related to health service use [17, 21–23]. Additionally, we figured out the demand for HBPC and relevant determinants for that demand depending on the report type (self-report or proxy report) for two reasons. First, we considered the proxy responses not only as a representative of disabled persons but also as primary formal/informal caregivers' perspectives who would have unique opinions about HBPC. Second, proxy-reported respondents are more likely to have difficulties in communication or cognitive function, and high care needs than self-reported respondents, even with the same severity of disability.

## Methods

### Study design and data

We analyzed the data from the population-based survey conducted in South Korea. The data was designed with quota random sampling based on the population of PWDs in each district (city and county) across Gyeonggi province, which contains the largest number of PWDs residing in South Korea (> 20% of the total PWDs), to evaluate the health and healthcare accessibility of the disabled in the community. The data was collected through the mobile phone-based survey of 1,140 PWDs between March and June 2021. This survey allowed proxy responses by their caregivers to collect the answers of those who could not respond via a phone survey. Of the 1,140 data respondents, we limited our sample to 755 PWDs aged ≥ 20 years who reported that they demanded the PHC pilot program regardless of a preference for HBPC. Because the dependent variable, demand for HBPC, was asked only the respondents who answered that they wanted the PHC pilot program for PWDs, and most respondents aged < 20 years belonged to the proxy-reported group.

### Measurement and variables

Demand for HBPC was regarded as a “home-based care” response to the question “Which way would you like to receive the PHC pilot services for PWDs: home-based care or office-based care or doesn’t matter (either home-based or office-based care)?”. If the respondents answered that they preferred either type of care, it could still be interpreted as a demand for HBPC, however we strictly limited our responses to those preferring only home-based care.

Characteristics of PWDs and health-related variables were categorized into predisposing, enabling, and need factors by modifying Andersen’s behavioral model of health service use. Demographic characteristics, such as age (20–39, 40–64, and ≥ 65 years), gender, marital status, education level, and living arrangement (e.g., living alone), were categorized as predisposing factors. Enabling factors included income level, occupational status, residential area, type of public health insurance, support status, use of any health services (home-based nursing or community-based rehabilitation from public health centers, health promotion program), use of health information sources, and awareness of the PHC pilot program (“yes” response to the question “Do you know about the PHC pilot project for PWDs?”), homebound status (going out less than once per week and needing help from others for most activities of daily living [ADLs]). To assess homebound status, we mixed the idea from the two prior studies for the conciseness and validity; one idea used; homebound regarded as “severe” or “extreme or cannot do” response to the question “In the past 30 days, how much difficulty did you have in leaving home?” None/mild/moderate/severe/extreme or cannot do” [24] and the other idea used; “how many days they left their home during the previous week?” [25]. Using the mixed idea, we can focus on difficulty and low frequency of going out caused by limitation of activities or care deficits.

Need factors included chronic disease, need for assistance with ADLs (defined as “no need for assistance” only if the PWD could do everything alone), subjective health status, severity of disability, subjective depressive status, unmet needs for medical care with financial or availability barriers (transportation problems or lack of adequate medical facilities), and disability type (i.e., external physical, mental and developmental, or internal organ). In Korea, based on the Welfare of Disabled Persons Act, registration and grading systems for PWDs categorized the officially registered disability type; 1) external physical: physical disabilities, brain lesion disorders, visual impairment, hearing impairment, language disabilities, facial disfigurement, 2) mental and developmental: intellectual disabilities, autistic disorder, mental disabilities, 3) internal organ: renal impairment, cardiac impairment, respiratory impairment, hepatic impairment, intestinal or urinary fistula, and epilepsy disorder [26].

Variables for additional analysis about the demand for HBPC depending on the report type (self-report or proxy report) in the severe disability group, the potential service users, were reconsidered to examine our hypothesis concerning the relevant factors that affect proxy-reported respondents who were more likely to have difficulties in communication or cognitive function, and high care needs than self-reported respondents even with the same severity of disability, to want HBPC. Based on modified Andersen’s behavioral model of health service use, variables associated with high care needs were selected; age, gender, marital status, income, homebound status, chronic disease, disability type, assistance in ADLs, and unmet medical care needs were included, while self-reported depressive symptoms, awareness of the PHC pilot program, and subjective health status that require reporting emotional feelings or thoughts were excluded since the proxy reports cannot adequately substitute for self-reports [27, 28].

### Statistical analysis

We conducted a descriptive analysis to identify overall characteristics and HBPC demand. The χ^2^ test or Fisher's exact test was used to compare the demand for HBPC according to the respondents’ characteristics. Second, multivariate analysis using a logistic regression model was performed to estimate the adjusted odds ratios (ORs) for the association between the demand for HBPC and health service use factors. When the mean variance expansion index (VIF) between all variables is ≥ 10 [29, 30] or the tolerance value is < 0.2, multicollinearity problems affecting the explanatory power and confidence interval (CI) of the model may occur [31]. Therefore, we confirmed the absence of multicollinearity by calculating the VIF and tolerance of the variables (VIF 1–2 for each variable; tolerance, 0.50–0.96; mean VIF, 1.36). All analyses were performed using the STATA 17 statistical package.

### Ethics statement

The present study protocol was reviewed and approved by the institutional review board of B Hospital (approval No. B-2101-661-304).

## Results

Among the 755 respondents, 22% (*n* = 166) reported a demand for HBPC (Table 1). Participants who were aged ≥ 65 years, female, and lower in education level reported a higher demand for HBPC, while fewer married participants demanded HBPC.

**Table 1:** Demographic and health related variables according to “Demand” on the HBPC

Variables		All (*n* = 755)	Demand		
			**Yes (*n* = 166)**	**No (*n* = 589)**	***P value***
**Predisposing factors**	**Age (years)**				
	20–39	152 (100.0)	26 (17.1)	126 (82.9)	
	40–64	430 (100.0)	80 (18.6)	350 (81.4)	< 0.001***
	65 +	173 (100.0)	60 (34.7)	113 (65.3)	
	**Gender**				
	Male	509 (100.0)	99 (19.5)	410 (80.6)	0.015*
	Female	246 (100.0)	67 (27.2)	179 (72.8)	
	**Marital status**				
	Married	415 (100.0)	70 (16.9)	345 (83.1)	< 0.001***
	Unmarried	340 (100.0)	96 (28.2)	244 (71.8)	
	**Education**				
	< High school (None, primary, middle)	128 (100.0)	53 (41.4)	75 (58.6)	< 0.001***
	High school	299 (100.0)	53 (17.7)	246 (82.3)	
	College, University	328 (100.0)	60 (18.3)	268 (81.7)	
	**Living Arrangement**				
	Live Alone	114 (100.0)	22 (19.3)	92 (80.7)	0.452
	Live with others	641 (100.0)	144 (22.5)	497 (77.5)	
**Enabling factors**	**Income** ^a^				
	Low	308 (100.0)	93 (30.2)	215 (69.8)	< 0.001***
	Medium	315 (100.0)	42 (13.3)	273 (86.7)	
	High	132 (100.0)	31 (23.5)	101 (76.5)	
	**Occupational status**				
	Currently employed	430 (100.0)	62 (14.4)	368 (85.6)	< 0.001***
	Unemployed	325 (100.0)	104 (32.0)	221 (68.0)	
	**Residential area**				
	Urban	456 (100.0)	102 (22.4)	354 (77.6)	0.884
	Suburban	282 (100.0)	61 (21.6)	221 (78.4)	
	Rural	17 (100.0)	3 (17.7)	14 (82.4)	
	**Public health insurance**				
	Health insurance	670 (100.0)	148 (22.1)	522 (77.9)	0.848
	Medical aid	85 (100.0)	18 (21.2)	67 (78.8)	
	**Homebound Status**				
	Homebound	83 (100.0)	47 (56.6)	36 (43.4)	< 0.001***
	Non-homebound	672 (100.0)	119 (17.7)	553 (82.3)	
	**Support status**				
	None	337 (100.0)	54 (16.0)	283 (84.0)	< 0.001***
	Care giver (informal, formal)	418 (100.0)	112 (26.8)	306 (73.2)	
	**Utilization of public health services**				
	Experienced	197 (100.0)	63 (32.0)	134 (68.0)	< 0.001***
	Not experienced	558 (100.0)	103 (18.5)	455 (81.5)	
	**Health information sources**				
	None	128 (100.0)	21 (16.4)	107 (83.6)	0.256
	Health service center	90 (100.0)	22 (24.4)	68 (75.6)	
	Welfare center/ Government office	125 (100.0)	34 (27.2)	91 (72.8)	
	Family, Friend Neighborhood	80 (100.0)	20 (25.0)	60 (75.0)	
	Mass media	332 (100.0)	69 (20.8)	263 (79.2)	
	**Awareness of the primary healthcare pilot project**				
	No	671 (100.0)	142 (21.2)	529 (78.8)	0.122
	Yes	84 (100.0)	24 (28.6)	60 (71.4)	
**Need factors**	**Chronic disease**				
	None	198 (100.0)	27 (13.6)	171 (86.4)	0.001**
	Yes	557 (100.0)	139 (25.0)	418 (75.0)	
	**Assistance in ADLs**				
	Need	320 (100.0)	108 (33.8)	212 (66.3)	< 0.001***
	Not need	435 (100.0)	58 (13.3)	377 (86.7)	
	**Subjective health status**				
	Good	112 (100.0)	16 (14.3)	96 (85.7)	0.001**
	Moderate	333 (100.0)	62 (18.6)	271 (81.4)	
	Poor	310 (100.0)	88 (28.4)	222 (71.6)	
	**Severity of Disability**				
	Severe	334 (100.0)	93 (27.8)	241 (72.2)	0.001**
	Mild	421 (100.0)	73 (17.3)	348 (82.7)	
	**Depressive Status**				
	None	357 (100.0)	54 (15.1)	313 (84.9)	< 0.001***
	Depressive	398 (100.0)	112 (28.1)	286 (71.9)	
	**Disability type**				
	External physical	600 (100.0)	136 (22.7)	464 (77.3)	0.171
	Mental, Developmental	84 (100.0)	12 (14.3)	72 (85.7)	
	Internal organ	71 (100.0)	18 (25.4)	53 (74.7)	
	**Unmet medical need-financial barrier**				
	None	698 (100.0)	156 (22.4)	542 (77.7)	0.400
	Unmet need	57 (100.0)	10 (17.5)	47 (82.5)	
	**Unmet medical need-availability barrier (Lack of clinics/hospitals, transportation problems)**				
	None	661 (100.0)	121 (18.3)	540 (81.7)	< 0.001***
	Unmet need	94 (100.0)	45 (47.9)	49 (52.1)	

*P* value. *P < 0.05, **P < 0.01, ***P < 0.001

Income ^a^ -low: lower than ₩1,000,000 ($752), medium: higher than ₩1,000,000, lower than ₩3,000,000 ($2,254), high: higher than ₩3,000,000 ($2,254)

PWDs who were unemployed and had experience of other health services from a public health center reported a higher demand for HBPC. High- or low-income PWDs reported a higher demand for HBPC, compared to medium-income individuals. A greater proportion of home-bound subjects (56.6%) reported a higher demand for HBPC, compared with that of non-homebound subjects. The participants with informal or formal caregiver (26.8%) expressed a higher demand for HBPC than those who without caregivers. Among the need factors, significant differences between PWDs demanding and not demanding for HBPC existed in chronic disease, assistance in ADLs, subjective health status and depressive status. More participants with severe disability (27.8%) significantly demanded HBPC, compared to those with mild disability, while there was no significant difference in disability type between PWDs demanding and not demanding for HBPC. Among all PWDs with unmet healthcare needs, 47.9% showed a greater demand for HBPC than those without.

Table 2 presents that six variables were predictors of the demand for HBPC. PWDs aged ≥ 65 years were more likely to report a demand for HBPC than PWDs aged < 20 years. Compared to married PWDs, unmarried PWDs reported a higher demand for HBPC. High-income level, home-bound status, and awareness of the PHC pilot program were associated with a higher demand for HBPC. Among the need factors, the need for assistance with ADL was correlated with a greater demand for HBPC.

**Table 2:** Multiple logistic regression of factors affecting “Demand” on the HBPC depending on the severity of disability

Variables		Demand OR (95% CI)		
		**All (*n* = 755)**	**Severe disability (*n* = 334)**	**Mild disability (*n* = 421)**
**Predisposing factors**	**Age (years)**			
	20–39	ref	ref	ref
	40–64	1.35 (0.72 – 2.55)	1.69 (0.64 – 4.50)	1.09 (0.43 – 2.74)
	65 +	2.29 (1.01 – 4.82)*	3.42 (1.03 – 11.31)*	1.89 (0.65 – 5.49)
	**Gender**			
	Male	ref	ref	Ref
	Female	1.15 (0.75 – 1.76)	1.14 (0.61 – 2.12)	1.31 (0.68 – 2.49)
	**Marital status**			
	Married	ref	ref	ref
	Unmarried	1.98 (1.21 – 3.24)**	2.16 (1.05 – 4.45)*	2.06 (0.98 – 4.32)
	**Education**			
	College, University	ref	ref	Ref
	High school	0.77 (0.48 – 1.24)	0.67 (0.33 – 1.35)	0.82 (0.40 – 1.69)
	< High school (None, primary, middle)	1.48 (0.81 – 2.70)	1.04 (0.44 – 2.45)	2.17 (0.85 – 5.53)
	**Living Arrangement**			
	Live with others	ref	ref	ref
	Live Alone	0.62 (0.33 – 1.14)	0.85 (0.36 – 2.03)	0.42 (0.16 – 1.12)
**Enabling factors**	**Income** ^a^			
	Low	ref	ref	ref
	Medium	0.76 (0.44 – 1.32)	0.41 (0.17 – 0.98)*	1.08 (0.47 – 2.48)
	High	2.53 (1.25 – 5.12)*	2.05 (0.66 – 6.35)	2.97 (1.04 – 8.45)*
	**Occupational status**			
	Currently employed	ref	ref	ref
	Unemployed	1.49 (0.88 – 2.52)	1.88 (0.83 – 4.26)	1.11 (0.49 – 2.51)
	**Residential area**			
	Urban	ref	ref	ref
	Suburban	0.78 (0.52 – 1.19)	0.70 (0.38 – 1.30)	0.78 (0.42 – 1.46)
	Rural	0.53 (0.10 – 2.77)	0.11 (0.01 – 1.63)	3.44 (0.35 – 33.86)
	**Public health insurance**			
	Health insurance	ref	ref	ref
	Medical aid	0.61 (0.31 – 1.19)	0.75 (0.32 – 1.79)	0.35 (0.09 – 1.41)
	**Homebound Status**			
	Non-homebound	ref	ref	ref
	Homebound	1.90 (1.04 – 3.49)*	1.58 (0.74 – 3.34)	2.00 (0.55 – 7.33)
	**Support status**			
	Care giver (informal, formal)	ref	ref	ref
	None	1.47 (0.85 – 2.56)	1.52 (0.57 – 4.05)	1.59 (0.76 – 3.31)
	**Utilization of public health services**			
	Not experienced	ref	ref	ref
	Experienced	1.29 (0.82 – 2.05)	1.33 (0.71 – 2.48)	1.51 (0.69 – 3.31)
	**Health information sources**			
	None	ref	ref	ref
	Health service center	1.39 (0.64 – 2.99)	1.15 (0.35 – 3.76)	1.68 (0.54 – 5.19)
	Welfare center/ Government office	1.67 (0.82 – 3.40)	1.36 (0.48 – 3.84)	2.22 (0.75 – 6.57)
	Family, Friend Neighborhood	1.13 (0.51 – 2.50)	1.04 (0.33 – 3.26)	1.36 (0.41 – 4.48)
	Mass media	1.61 (0.88 – 2.95)	1.49 (0.59 – 3.77)	1.95 (0.82 – 4.64)
	**Awareness of the primary healthcare pilot project**			
	No	ref	ref	ref
	Yes	1.94 (1.06 – 3.54)*	0.98 (0.39 – 2.43)	3.25 (1.31 – 8.05)*
**Need factors**	**Chronic disease**			
	None	ref	ref	ref
	Yes	1.23 (0.73 – 2.08)	1.35 (0.61 – 2.97)	1.05 (0.50 – 2.21)
	**Assistance in ADLs**			
	Not need	ref	ref	ref
	Need	2.05 (1.14 – 3.70)*	1.99 (0.74 – 5.35)	2.25 (0.99 – 5.15)
	**Subjective health status**			
	Good	ref	ref	ref
	Moderate	1.27 (0.66 – 2.46)	1.63 (0.47 – 5.67)	1.19 (0.51 – 3.41)
	Poor	1.23 (0.60 – 2.55)	1.58 (0.43 – 5.78)	1.24 (0.45 – 3.41)
	**Disability type**			
	Not external physical (Mental, Developmental, Internal organ)	ref	ref	Ref
	External physical	1.24 (0.72 – 2.15)	1.75 (0.79 – 3.85)	0.88 (0.37 – 2.09)
	**Depressive Status**			
	None	ref	ref	ref
	Depressive	1.43 (0.90 – 2.26)	1.76 (0.86 – 3.61)	1.10 (0.57 – 2.11)
	**Unmet medical need-financial barrier**			
	None	ref	ref	ref
	Unmet need	0.64 (0.29 – 1.44)	1.44 (0.48 – 4.29)	0.28 (0.06 – 1.38)
	**Unmet medical need-availability barrier (Lack of clinics/hospitals, transportation problems)**			
	None	ref	ref	ref
	Unmet need	1.77 (1.00 – 3.13)	2.15 (1.02 – 4.50)*	1.36 (0.43 – 4.36)
	**Severity of Disability**			
	Not severe	ref		
	Severe	1.22 (0.76 – 1.96)		
Log likelihood		-328.12243	-148.87412	-163.25126
Pseudo R2		0.1749	0.2464	0.1593

Income ^a^ -low: lower than ₩1,000,000 ($752), medium: higher than ₩1,000,000, lower than ₩3,000,000 ($2,254), high: higher than ₩3,000,000 ($2,254)

*OR* odds ratio, *CI* confidence interval, *P* value. *P < 0.05, **P < 0.01

We conducted a subgroup analysis since the beneficiaries of the current HBPC services under the PHC pilot program are confined to people with registered severe disabilities. Among people with severe disabilities, respondents who were aged ≥ 65 years (OR, 3.42; 95% CI, 1.03–11.31), unmarried (OR, 2.16; 95% CI, 1.05–4.45), and with unmet needs for medical care with availability barriers (e.g., transportation problems) (OR, 2.15; 95% CI, 1.02–4.50) reported a higher demand for HBPC (Table 2).

In the severe disability group, 41.62% of the responses were proxy-reported. 91.39% of them reported that they needed assistance with ADL; in contrast, 45.34% of self-reported respondents with severe disability reported the need for assistance with ADL (χ2 = 85.065; *P* < 0.001). Moreover, all of the proxy-reported respondents except one responded that they had formal or informal caregivers, while 54.66% of self-reported respondents reported that they had caregivers (χ2 = 92.023; *P* < 0.001). There was a big difference in the demand for HBPC according to the report type, possibly resulting from a different perspective on HBPC between PWDs and caregivers. Only 19.49% of self-reported respondents demanded for HBPC; in contrast, 39.57% of proxy-reported respondents demanded for HBPC (Table 3). More PWDs without any caregivers reported a demand for HBPC among the self-reported group compared to those with either formal or informal caregivers. Among the proxy-reported group, more PWDs with formal caregivers demanded for HBPC, compared to those with informal caregivers (Table 4).

**Table 3:** Demands on the HBPC according to the self-report and proxy-report (caregiver-report)

	**Severe disability**			**Mild disability**		
**Home-based primary care**	**Self-report (*n* = 195)**	**Proxy-report (*n* = 139)**	***P value***	**Self-report (*n* = 377)**	**Proxy-report (*n* = 44)**	***P value***
Not need	157 (80.51)	84 (60.43)	< 0.001***	320 (84.88)	28 (63.64)	< 0.001***
Need	38 (19.49)	55 (39.57)		57 (15.12)	16 (36.36)	

*P* value. ***P < 0.001

**Table 4:** Caregivers type among the participants who want HBPC according to the self-report and proxy-report (caregiver-report)

	**Severe disability (*n* = 334)**					
	**HBPC want**			**HBPC not want**		
**Caregivers**	**Self-report (*n* = 38)**	**proxy-report (*n* = 55)**	***P value***	**Self-report (*n* = 157)**	**proxy-report (*n* = 83)**	***P value***
None	16 (42.11)	0 (0.00)	< 0.001***	69 (43.95)	0 (0.00)	< 0.001***
Family	11 (28.95)	22 (40.00)		66 (42.04)	44 (53.01)	
Neighborhood	3 (7.89)	2 (3.64)		11 (7.01)	8 (9.64)	
Formal	8 (21.05)	31 (56.36)		11 (7.01)	31 (37.35)	

*P* value. ***P < 0.001

Only unmarried PWDs (divorced, separated, widowed, or never married) expressed a greater need for HBPC compared to married PWDs among the self-reported group with severe disability; in contrast, PWDs with external physical disabilities, or with unmet needs for medical care with availability barriers reported a higher demand for HBPC among the proxy-reported group (Table 5).

**Table 5:** Multiple logistic regression of factors affecting “Demand” on the HBPC depending on the self-report and proxy-report (caregiver-report)

Variables		Severe disability OR (95% CI)	
		**Self-report (*n* = 195)**	**proxy-report (*n* = 139)**
**Predisposing factors**	**Age (years)**		
	20–39	ref	ref
	40–64	5.08 (0.85 – 30.37)	1.73 (0.41 – 7.40)
	65 +	4.66 (0.55 – 39.15)	3.91 (0.81 – 18.83)
	**Gender**		
	Male	ref	ref
	Female	1.40 (0.57 – 3.43)	1.29 (0.49 – 3.40)
	**Marital status**		
	Married	ref	ref
	Unmarried	2.64 (1.03 – 6.75)*	2.19 (0.66 – 7.27)
	**Education**		
	College, University	ref	ref
	High school	0.71 (0.30 – 1.70)	0.52 (0.13 – 2.08)
	< High school (None, primary, middle)	1.11 (0.33 – 3.75)	0.38 (0.12 – 1.24)
	**Living Arrangement**		
	Live with others	ref	ref
	Live Alone	0.96 (0.34 – 2.68)	1.08 (0.18 – 6.42)
**Enabling factors**	**Income**^**b**^		
	Low	ref	ref
	High	0.51 (0.18– 1.47)	0.40 (0.08 – 1.92)
	**Occupational status**		
	Currently employed	ref	ref
	Unemployed	1.65 (0.56 – 4.85)	1.81 (0.44 – 7.45)
	**Residential area**		
	Urban/suburban	ref	ref
	Rural	1.25 (0.53 – 2.91)	1.08 (0.40 – 2.92)
	**Public health insurance**		
	Health insurance	ref	ref
	Medical aid	0.43 (0.12 – 1.54)	0.93 (0.27 – 3.28)
	**Homebound Status**		
	Non-homebound	ref	ref
	Homebound	1.00 (0.22 – 4.65)	1.50 (0.54 – 4.19)
	**Utilization of public health services**		
	Not experienced	ref	ref
	Experienced	0.87 (0.37 – 2.05)	1.76 (0.70 – 4.44)
	**Health information sources**		
	None	ref	ref
	Health service center	0.72 (0.17 – 3.07)	1.56 (0.17 – 14.32)
	Welfare center/ Government office	1.15 (0.30 – 4.38)	1.75 (0.30 – 10.18)
	Family, Friend Neighborhood	0.39 (0.06 – 2.55)	2.78 (0.46 – 16.78)
	Mass media	0.73 (0.23 – 2.34)	4.07 (0.81 – 20.47)
**Need factors**	**Chronic disease**		
	None	ref	ref
	Yes	1.73 (0.59 – 5.06)	0.97 (0.26 –3.63)
	**Assistance in ADLs**		
	Not need	ref	ref
	Need	1.73 (0.69 – 4.33)	2.26 (0.17 – 29.35)
	**Disability type**		
	Not external physical (Mental, Developmental, Internal organ)	ref	ref
	External physical	0.99 (0.31 – 3.13)	4.12 (1.29 – 13.18)*
	**Unmet medical need-financial barrier**		
	None	ref	ref
	Unmet need	0.88 (0.19 – 4.11)	2.08 (0.38 – 11.43)
	**Unmet medical need-availability barrier (Lack of clinics/hospitals, transportation problems)**		
	None	ref	ref
	Unmet need	1.05 (0.31 – 3.51)	3.59 (1.22 – 10.57)*
Log likelihood		-83.960061	-64.308174
Pseudo R2		0.1270	0.3107

Income ^b^ -low: lower than ₩1,000,000 ($752), high: higher than ₩1,000,000 ($752)

*OR* odds ratio, *CI* confidence interval, *P* value. *P < 0.05

## Discussion

Our community-based cross-sectional survey showed that 22% of the PWDs required the HBPC and 34.7% of PWD aged 65 years and above demanded the HBPC. Older adults with disability, homebound status, need for assistance with ADL were associated with demands for the HBPC. According to the characteristics and report-type, the demand for HBPC varied. Our findings are intended to provide evidence for need-based demands to expand the HBPC model for PWDs with barriers to accessing to office-based care.

The demand for HBPC might be underestimated due to our strict definition. Preferring either home-based or office-based care was interpreted as having no demands for HBPC, because the responders might not be strong potential consumers of HBPC with less barriers in accessing traditional primary care, such as office-based care. Although this result may be underestimated because of our strict definition of demand, the demands of older adults with disability were at similar levels to that from other studies [32], which defined HBPC demands as a “yes” response to the question “Do you want a doctor's home visiting service when you need help?”, of which 39.3% of Korean older adults need HBPC. Since people with long-term disabilities have problems with premature aging and late effects, new health problems deriving from the chronic impairment, primary care physicians need to pay attention to the life course of people with severe disabilities in the community [33, 34].

Homebound respondents may demand for HBPC since they have difficulty in accessing office-based care as they have poorer health conditions and are more functionally dependent. A homebound status strongly predicted the acquisition of a disability in older populations, which showed that functional limitations may restrict contacts with the healthcare system, such as office-based care, and cause functional deterioration, resulting in a vicious circle [35]. Even though the indicators of functional limitation, such as severity of disability and need for assistance with ADL, were used for adjustment, homebound status was still significantly associated with the demands for HBPC. It can be explained that homebound respondents tend to demand for HBPC, expecting that their psychological problems would be managed. A homebound population is more prone to depression [36], as those with disability can be more socially isolated by physical and environmental barriers [37]. Our finding that unmarried respondents with severe disability were most likely to demand for HBPC also may be due to the absence of a spouse to offer emotional support or primary care. This is consistent with prior studies that subjective loneliness affected the need for physician home visits in Korean older adults [32] and care deficit was greatest in older men having disabilities without a spouse [38]. Moreover, in Korea, giving spouse care is common; spouse caregivers have the highest prevalence among informal caregivers [39]. HBPC can contribute to improving quality of life, including mental health and social support to deal with social isolation [40].

The proxy-reported group showed a higher demand for HBPC than the self-reported group. It can be interpretated in two aspects; First, though having severe disability, self-reporting respondents might have better functional status than PWDs in proxy-reported group. In South Korea, the Korea National Disability Registration System (KNDRS) has been used to deliver most public or social services to PWDs by the government. While KNDRS has focused on a medical or impairment approach with benefits of objective measures to assess disability and comfortable means which can be linked to national health data, it has limitations for delivering personalized service that it does not reflect other aspects of disability, such as socioeconomic status and functional status [41]. In this context, proxy respondents with unmet care needs caused by complex determinants such as socioeconomic factors, disability type, and eligible care resources could be more likely to want HBPC. Especially, our findings that PWDs with external physical disabilities, or with unmet medical need due to the transportation problems had higher demand for HBPC in the proxy-reported group strengthen the facts that care deficits and high care needs are core determinants of demand for the HBPC.

Second, these results attributed from the differences in the perspectives of HBPC between PWDs and their caregivers. The demand for HBPC in the proxy-reported group, might be deemed as a caregivers’ perception. Some PWDs could hesitated at receiving HBPC because doctor’s appointments were the only opportunities, they left their homes [41]. However, in that case, caregivers could feel stressed and burdened since they meet challenges in accompanying PWDs who need physical assistance to hospitals such as and transportation barriers [42] or missing their working time. Moreover, since office-based care involved difficulties with making timely appointments and long wait times [41], access was markedly impaired in persons for whom caregivers were not consistently available [4]. Similarly, a previous study reported that HBPC could alleviate the caregiver burden [6]. However, a careful interpretation is needed since the proxy responses couldn’t be complete perspectives of caregivers. In our study, seeds have been found that can be left to future research for proxy responses and caregivers’ perspectives.

An interesting finding in our study was that PWDs with the formal caregivers expressed a higher demand for HBPC than family caregivers. Though formal caregiving system to PWDs, a care gap for people with severe disability exists due to a shortage of formal caregivers available to shoulder the greater care burden [43]. Professional treatment and support is thought to reduce the probability of experiencing care stress by reducing care time and relieving responsibility and pressure for healthcare [44]. As the number of PWDs living alone without grown children or a spouse to provide care is increasing, demands for formal caregiving will increase. HBPC can alleviate the caregiver burden and patients’ care deficits for quality of life.

There are several limitations in this study. First, this study might have selection bias, because the data used in this study were focused on regional stratification without considering gender, age, or type of disability in relation to the sample composition. Second, data were collected through a mobile-based survey during the coronavirus disease 2019 pandemic; in effect, the survey was not accessible to some PWDs due to disability characteristics or illiteracy. We used the data including the proxy-reports which allowed parents or caregivers to respond on behalf of PWDs to address the limited accessibility of a mobile-based survey. Third, we indirectly assessed the caregivers’ demand using the proxy-report. It means that there is a possibility which our findings were not the direct perspectives of caregivers but only a representative of perspectives of PWDs. Since caregiving and caregiver burden have become increasingly important social issues, further work is needed to identify the perspectives of caregivers as much as those of PWDs.

## Conclusions

Characteristics related to poor accessibility to office-based care, such as homebound patient status, need for assistance with ADLs, and unmet needs for medical care due to availability barriers predicted the demand for HBPC on the perspectives of PWDs. This study provides an opportunity to show that HBPC does not derive from the medical demands of the users themselves, but rather a strong background of relative lack of care for the reality of difficulty in getting out of the house or in outpatient care. The HBPC model is one way to address barriers to accessing healthcare for PWDs and support aging in place and deinstitutionalization [45, 46]. HBPC is associated with patients’ preference for home death since HBPC might increase the satisfaction of patients and reduce caregiver burden [46]. Most HBPC programs have been based on perspectives of medical staffs focusing on the “need” for HBPC; however, the demand for HBPC is a key attribute of patient-centered care. Through our results, HBPC providers can be provided with evidence for potential service consumers. Although HBPC has expanded with higher value [5], HBPC is still underused due to poor and maldistributed supply structures [17, 47]. From a systemic perspective, the supply should coincide with need-based demands in the most efficient and equitable way for a given set of resource constraints [48]. We suggest that matching HBPC supply with HBPC demand is necessary and will contribute to alleviating the healthcare access barriers for aging populations with severe disabilities, as well as caregiver burden.

## Data Availability

The datasets used during the current study available from the corresponding author on reasonable request.
